# Pregnant individuals perspectives towards receiving COVID-19 vaccination during their pregnancy: an in-depth qualitative study

**DOI:** 10.3389/fpubh.2024.1415548

**Published:** 2024-08-21

**Authors:** Sanne J. M. Zilver, Anna L. Rietveld, Noralie N. Schonewille, Petra C. A. M. Bakker, Birit F. P. Broekman, Elisabeth van Leeuwen, Christianne J. M. de Groot

**Affiliations:** ^1^Amsterdam Reproduction and Development Research Institute, Amsterdam, Netherlands; ^2^Department of Obstetrics and Gynaecology, Amsterdam UMC Location Vrije Universiteit and University of Amsterdam, Amsterdam, Netherlands; ^3^Department of Psychiatry and Medical Psychology, OLVG, Amsterdam, Netherlands; ^4^Department of Psychiatry, Amsterdam UMC Vrije Universiteit, Amsterdam, Netherlands; ^5^Amsterdam Public Health, Mental Health Program, Amsterdam, Netherlands

**Keywords:** pregnancy, pregnant individuals, COVID-19, vaccine hesitancy, SARS-CoV-2

## Abstract

**Introduction:**

Pregnant individuals have an increased risk of severe illness from coronavirus disease 2019 (COVID-19) infection. Vaccination is an effective strategy to prevent severe illness and complications for pregnant individuals. Pregnant individuals are often excluded from research and remain hesitant to receive vaccination against COVID-19. It is pivotal to study factors related to vaccine uptake and hesitancy among pregnant individuals. We studied barriers and facilitators for pregnant individuals choice and motivation regarding vaccination against COVID-19 during pregnancy to aid future pregnant individuals in their decision to vaccinate against various infectious agents.

**Methods:**

In this qualitative study, pregnant individuals were interviewed between October 2021 and January 2022 using a semi-structured approach. A topic list was used to explore their feelings, perceptions and ideas regarding vaccination against COVID-19 during pregnancy. Interviews were transcribed verbatim and thematic analyses was performed using MAX QDA.

**Results:**

After nine interviews, saturation was reached. Three main themes were identified that influenced pregnant individuals choice and motivation regarding vaccination: health consequences, ambiguity of information and societal motivation. Health consequences mainly concerned the effect for their offspring, and the unknown long-term effects of COVID-19 vaccination. The advice from the Dutch institute for Public Health and Environment changed from not vaccinating pregnant individuals after release of the developed vaccine, to routinely vaccinating all pregnant individuals after research data were available from the United States of America (USA). This change of policy fuelled doubt and confusion for vaccination. Arguments in favor of vaccination from the social perspective were specific behaviour rules and restrictions due to the pandemic. E.g. without vaccination people were unable to travel abroad and having to take a COVID-19 test every time entering a public place.

**Conclusion:**

Pregnant individuals need clear, unambiguous information concerning health consequences, short- and long-term, particularly for their offspring, in the decision-making process regarding COVID-19 vaccination. Additionally, the societal perspective needs to be addressed. Besides the aforementioned themes, general counselling should focus on misperceptions of vaccine safety and the role of misinformation which are also important in the non-pregnant population. This study underlines the importance of including pregnant individuals in research programs to obtain specific information targeted to their needs.

## 1 Introduction

The COVID-19 pandemic has an enormous global impact, and therefore is different from other recent infectious disease outbreaks (1–3). Disease burden, social isolation and distancing, loss of work, mental health problems and economic implications were unique in intensity, abruptness and severity and many of these still continue to have an effect on society.

Vaccines against the virus that causes COVID-19, severe acute respiratory syndrome coronavirus 2 (SARS-CoV-2), were developed in a very fast and novel manner, enabling protection against the sequelae of an infection with the virus, especially the mRNA vaccines. More than 70% of the general population accepted the vaccine (4). During the pandemic, pregnant individuals were more hesitant to receive a vaccination compared to the general population (5–8). This is particularly important, since pregnant individuals were more vulnerable to complications from COVID-19. Although severe COVID-19 is uncommon, compared to non-pregnant individuals, pregnant individuals showed higher rates of intensive care unit (ICU) admission, invasive ventilation, extracorporeal membrane oxygenation (ECMO) and higher mortality rates (9). Furthermore, pregnant individuals with severe COVID-19 have higher rates of iatrogenic preterm birth, leading to higher rates of neonatal intensive care unit (NICU) admissions (10). When comparing pregnant individuals with COVID-19 to pregnant individuals without COVID-19, severe neonatal complications are higher in pregnant individuals with COVID-19 (11). Fajardo-Martinez et al. found neurodevelopmental delay in children age 5–30 months who were exposed to maternal Sars-CoV-2 *in utero* (12).

Initially, in 2020, pregnant individuals were excluded from phase 2 and 3 COVID-19 vaccine trials, due to safety regulations (13). However, in several countries, e.g., in the USA, pregnant individuals were able to receive the vaccine (14). As a result, data on the safety and effectivity of the vaccine became rapidly available. These data and data from individuals who inadvertently became pregnant during the COVID-19 vaccine clinical trials showed similar immunogenicity for pregnant individuals compared to non-pregnant individuals (15) and no specific risks, including side effects or adverse birth outcomes (16, 17). This resulted, at first, in an advice for pregnant individuals with underlying medical health conditions or an occupation with high risk of contact with SARS-CoV-2, to get vaccinated (18). When more data became available, the (international) advice changed from vaccination for this selection of pregnant individuals to vaccination of all pregnant individuals (19). In Netherlands, pregnant individuals were advised to receive a mRNA vaccine (Pfizer BioNTech BNT162b2 and Moderna mRNA-1273). Additionally latter data showed maternal COVID-19 vaccination was associated with lower risk of COVID-19 related hospitalization in infants <6 months of age (20). However, pregnant individuals remained hesitant to receive a COVID-19 vaccine (21); with an estimated acceptance percentage of 49% worldwide (4, 22).

Vaccine hesitancy has been defined as “a delay or refusal of vaccination despite the availability.” There are several determinants that influence vaccine hesitancy which vary across time, between diseases, vaccines and communities (23). To investigate what determinants are of influence, two commonly used models are available to explain vaccine hesitancy. The first model is the 3 C’s model, comprising of complacency, convenience and confidence (23). Within this model, complacency is the perceived notion that the risk of vaccine-preventable disease is low and therefore vaccination is not a necessary preventive measure. Convenience comprehends factors such as geographical accessibility, affordability, physical availability and the ability to understand in terms of health literacy. Confidence is defined as trust in the safety and effectiveness of vaccines and the system that delivers them. This includes the competence of health care workers providing the vaccine. The Health Believe Model (HBM) is another concept that is used to explore and explain the rationale behind vaccine hesitancy, as it is a widely used to predict health behaviour (24). The HBM relies on self-efficacy in explaining health behaviour and perceived susceptibility, severity, benefits, and barriers. The HBM is based on the hypothesis that the response to a health threat is determined by a person’s perceived severity of the threat and susceptibility to the threat. Engaging in health protective behaviour is determined by the estimated benefits of the protective behaviour and potential barriers against the behaviour.

Predictors of COVID-19 vaccine acceptance in pregnant individuals among different studies include: advanced maternal age, occupational status (employed individuals were more likely to receive vaccination), higher educational level, white race, having a previous influenza vaccination, third trimester of pregnancy, comorbidities, knowledge about COVID-19 (25–28). During the COVID-19 pandemic, an additional and unaddressed issue possibly contributing to vaccine hesitancy in pregnant individuals was physician hesitancy to recommend the vaccine to pregnant individuals (29).

Due to the unique challenges and changes during the COVID-19 pandemic, we hypothesize that different and perhaps unique factors can be identified in the decision-making process regarding vaccination against COVID-19 during pregnancy. This knowledge is important in the perspective of upcoming diseases and necessity of newly developed vaccines (30, 31).

Therefore, the aim of this qualitative study is to explore barriers and facilitators for individuals in their decision regarding vaccination against a new virus, COVID-19, during pregnancy. The results of this study help to provide insight into which specific information will help pregnant individuals to make an informed decision about using newly developed vaccines and facilitates implementation of new vaccines in the near future during pregnancy.

## 2 Materials and methods

### 2.1 Design

We conducted a qualitative study of lived experiences of pregnant individuals regarding the decision-making process for vaccination against COVID-19. A qualitative design was chosen as we aimed to gain a comprehensive understanding of the barriers and facilitators that pregnant individuals encounter in the decision-making process. Semi-structured interviews were conducted that took a narrative approach. We hypothesized that pregnant individuals perspectives on vaccination are fuelled by their personal experiences and opinions, but also interact with the society surrounding them (32). A thematic analysis was chosen to analyse the data, as this methodology fits within the constructionist paradigm and holds space for an inquiry in the reality of participants.

### 2.2 Research team

A multidisciplinary research team ensured variety in perspectives. The research team included three gynaecologists, a psychiatrist, a gynaecologist in training and two PhD candidates in the field of pregnancy, mental health and COVID-19.

### 2.3 Setting and recruitment of participants

Pregnant individuals who received prenatal care in a tertiary care centre, Amsterdam University Medical Centre –location VUmc (Amsterdam UMC – VUmc) and low-risk pregnant individuals in midwifery practices in the Amsterdam area were asked to participate in the study. Purposive sampling was conducted to obtain a sample of pregnant individuals from diverse backgrounds including variations in maternal age, parity, country of birth, educational level and vaccination status. Pregnant individuals ≥18 years and with sufficient knowledge of the Dutch or English language were found eligible to participate in the study. Eligible individuals were initially approached by their health care professional and subsequently by the first author (SJMZ), to be informed about the study. Participants received an information letter, including an informed consent form. After informed consent was obtained, an interview date was scheduled. Participants could withdraw consent at any time. Participants were allowed to bring a support person to the interview. Interviews were scheduled until no new themes emerged from the data.

### 2.4 Data collection

**Figure 1:**
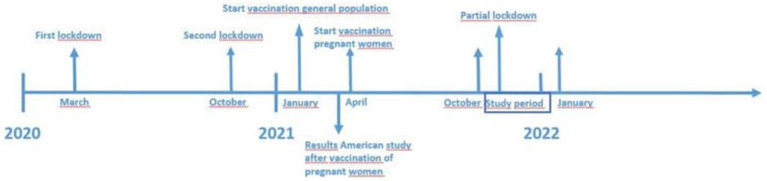
Timeline of lockdowns, start of vaccination and study period in Netherlands.

All data were collected between October 2021 and January 2022 (see Figure 1, timeline of lockdowns, vaccination availability and study period).

During a four-month period, semi-structured interviews took place by phone, video call or onsite at the Amsterdam UMC, depending on the preference of the participant. Onsite interviews were conducted in a private consultation room within the hospital’s outpatient clinic. The space was furnished with a desk, two chairs, and a small side table with informational pamphlets. This location was chosen to create a secure, calm environment for the participant. Despite the clinical setting, efforts were made to make the participant feel comfortable, with the interviewer offering drinks and explaining the purpose of the interview clearly at the outset. The choice of a hospital setting was intended and in consultation with the participant to make it convenient for the participant, who was already visiting the clinic for an appointment. Interviews lasted between 25 and 45 min and were conducted by the first author (SJMZ). All interviews were audio-recorded.

The self-developed interview guide (see Appendix 1) included a short list of background and general questions. To address the thoughts and ideas regarding vaccination and in particular COVID-19 vaccination, the interview started exploring subjects such as; general ideas on vaccination, prior vaccinations, thoughts about COVID-19 in general, experiences with COVID-19, sources of information on COVID-19, opinions of healthcare providers, relatives and friends on the subject of COVID-19 vaccination. Depending on the answers, more in-depth questions were asked to elaborate on the previous answers.

The study design and reporting adhered to Consolidated Criteria for Reporting Qualitative research (COREQ).

### 2.5 Data-analysis

Interviews were recorded and transcribed ad verbatim, followed by coding according to the principles of thematic analysis (33) using the qualitative analysis software MAX QDA. The analysis included a process of familiarizing with the data (by reading and rereading transcripts), coding and interpretation. Initially, open coding of all transcripts was performed by the first author (SJMZ). To enrich the variety of codes and to complement each other’s coding, two interviews were also coded by the second author (ALR) and three by the third author (NNS). Subsequently, interpretive coding was conducted by the research team in a group meeting offering diverse perspectives to enhance reliability. Themes were constructed from the selective codes as we extracted patterns of shared meaning from the data. The transcripts and findings were not returned to the participants for member checking.

### 2.6 Ethical considerations

The Medical Ethics Review Committee of VU University Medical Centre examined the study protocol (2021.0245). Official approval was not required. The protocol was in accordance with Dutch privacy regulations.

## 3 Results

A total of nine pregnant individuals participated in the study. During the recruitment period of the study, eight individuals declined to participate in the study, six of these individuals were not vaccinated. Their main reason for not participating was unwillingness to discuss the topic. The other two individuals were vaccinated and did not give a particular reason for not wanting to participate in the study. Out of the nine interviews, one interview was conducted in English.

Participant characteristics are shown in Table 1. Maternal age ranged from 28 to 40 years. Most of the participants were multiparous and had received care at the Amsterdam UMC. None of the interviewed individuals were in their first trimester, gestation ranged from 22 to 39 weeks.

**Table 1:** Background characteristics of the participants.

	Participants (*N* = 9)
**Age (mean, SD)**	34.8 (3.8)
**Country of birth**	
Belgium	1
Bulgaria	1
India	1
Morocco	1
Netherlands	4
Suriname	1
**Educational level**	
Secondary vocational education	3
Higher professional education	3
University education	3
**Marital status**	
Single	0
Living apart together	1
Living together	5
Married	3
**Previous treatment for psychological distress**	
Yes	4
No	4
Unknown	1
**Parity**	
Primipara	2
Multipara	7
**Gestational age in weeks (mean, SD)**	32.4 (6.2)
**Gestation**	
First trimester	0
Second trimester	3
Third trimester	6
**Prenatal care**	
Hospital	8
Midwifery practice	1
**Previous COVID-19 (self-reported)**	
Yes	**6**
No	**2**
Unknown	**1**
**Vaccinated against COVID-19**	
Yes	5
No	4

The bold values state the background characteristics.

After analyses, three main themes, related to decision-making regarding vaccination in pregnancy, were identified: (1) Health consequences, (2) Ambiguity of information, and (3) Societal motivation. Themes and subthemes are described in Table 2. Translated quotes support the themes. In addition, Table 3 shows the themes with the main explanations in vaccinated versus unvaccinated pregnant women.

**Table 2:** Overview of themes and subthemes.

Themes	Subthemes
Health consequences	Insecurity versus convinced of importance of vaccination Insecurity versus confidence in own health Consequences for offspring Consequences of illness
Ambiguity of information	Information provision Trust versus scepticism
Societal motivation	Altruism External motivation/government rules and restrictions

**Table 3:** Main themes with associated factors in vaccinated versus unvaccinated women.

	Vaccinated pregnant individuals (*n* = 5)	Unvaccinated pregnant individuals (*n* = 4)
Health consequences	Insecurity regarding own health during pregnancy and additional risks of COVID-19 infection	Unknown consequences for offspring
Ambiguity of information	Trust in information from health care providers/family	Sceptical about scarce research (at the time) Ambiguous information provision, with advise changing from not vaccinating pregnant individuals to routinely vaccinating pregnant individuals
Societal motivation	Unable to live a “normal” daily life Not being able to travel without vaccination	Feeling defensive, due to negative comments from society but this strengthened their own decision

### 3.1 Health consequences

Participants particularly expressed the importance of health for their unborn child. The unknown consequences of vaccination for their offspring were a main concern. This led to insecurities regarding the safety of vaccination versus the importance of vaccination, and also to insecurity versus confidence in their own health.

#### 3.1.1 Insecurity versus confidence in importance of vaccination

The majority of the participants recalled that prior to their pregnancy they were more open to receipt of a vaccine. However, participants perceived pregnancy to have a “special status”. This status concerns responsibility for the safety of the unborn child resulting in being extra careful with dietary restrictions and taking medication and, in addition, reluctance towards vaccination.

“Yes, 100%. If I had not been pregnant, I would have taken it immediately” (P2).

“Especially because during pregnancy you try really hard and do your best not to eat certain foods, take a lot of vitamins, you take all these things into consideration, and then you take a vaccine of which you do not know the long-term effects, this causes mixed feelings” (P1).

#### 3.1.2 Insecurity versus confidence in own health

Perceived physical health is an important theme but differs among participants. Some participants perceived their health as good and therefore were reluctant to choose vaccination.

“Because basically, I see myself as a healthy person” (P9).

Interestingly, another participant described the opposite. Explaining that the physical disability she already felt from being (heavily) pregnant made her unsure of what would happen if she would get a COVID-19 infection as well.

“Because now I really noticed that my breathing is high and shallow, and standing up, I immediately felt dizzy and out of breath, and I felt shortness of breath at night when I woke up. If this is how I feel just because of the pregnancy, then I do not think it will be okay if I have that (COVID-19) on top” (P4).

Other participants described having experienced COVID-19 in the past without severe symptoms being one of the reasons not the choose vaccination. As described above, vulnerability of health during pregnancy was an important theme. Whether or not that vulnerability was a reason for vaccination, differed between participants.

One participant described that she felt like her immune system was weaker and she was not sure if she wanted to receive a second vaccination during pregnancy, due to fear of feeling ill from the side effects of vaccination instead of from COVID-19 infection.

“Because I feel that my immunity is now a bit lower. I am afraid that those side effects from a possible second dose are more intense, and that I will get sick of that” (P4).

#### 3.1.3 Consequences for offspring

One of the items that was repeatedly mentioned during the interviews was not being able to know if there would be any adverse long-term effects of vaccination, in particular for their offspring. Some participants specifically expressed a fear for birth defects and long-term effects regarding for example infertility or attention disorders in their offspring.

“[…]I was only allowed an mRNA vaccination, but the long-term effects are just not known and of course you have those diethylstilbesterol (DES) children and the Softenon and you have more things that happened in the past with medication that turned out not to be such a good idea in the longer term, I just did not dare to take this” (P7).

“Yes, I find that hard to say. […]. I find it difficult if my child turns 30 and wants to have children of its own and then it turns out he or she is less fertile. I do not believe my child will come out with 5 arms, or that sort of thing, but maybe he or she will have an attention deficit disorder or something like that, and that that could be linked to the vaccine. I would find that very difficult” (P1).

#### 3.1.4 Consequences of illness

Another factor that influenced decision-making regarding COVID-19 vaccination are personal experiences. Some participants described how they felt when they had COVID-19.

“No, I just had a little muscle ache, just like when I have flu. Other than that, I have had no complaints” (P9).

“Well it was not pleasant, it was hard, but luckily I was at home. I did not have to go to the hospital. But I had to cough a lot and I have never been this sick in my life. Let me put it this way, it was much more than a flu” (P5).

Other participants described having family or friends who had COVID-19 without any severe symptoms or hospital admission. One participant shared that she had lost a family member due to complications of a COVID-19 infection and another participant described how someone she knew had delivered a baby while she had a COVID-19 infection and the aftermath of the infection.

“She really did not have enough oxygen to push and she was between life and death. After she gave birth, she also walked through her house with an oxygen tank and a baby on the other arm for months” (P7).

### 3.2 Ambiguity of information

A second theme focuses on the relation between information provision and decision-making regarding vaccination. In general, participants found it important that information is provided in a clear and concise way. Information was available from health care providers and through different platforms, such as television, newspapers, online and social media. Participants were having difficulties to determine which information was reliable.

#### 3.2.1 Information provision

Several participants have pointed out the difficulty of the vaccination advice changing over time from not (routinely) vaccinating pregnant women due to lack of data about the safety during pregnancy, to the advice of vaccinating all pregnant women.

“The conversations were overall good, the funny thing was, that people who got pregnant at the same time as me, were also advised not to get vaccinated […]. But after the government advised everybody to get vaccinated, people who have gotten pregnant since follow that advice” (P1).

In addition, participants searched for information online, ranging from scientific websites to news websites and to national vaccination advisory boards. Multiple participants expressed that information on social media did not influence their decision, because they did not take this information platform seriously.

“Through the internet, but I try to seek scientific articles, not Wikipedia or a pregnant person’s personal blog. I also looked for simple things such as: how long has the vaccine been used, how many years has it been tested, in which countries, all those sorts of things” (P1).

#### 3.2.2 Trust versus scepticism

Despite the fact that participants felt the decision regarding vaccination was a decision they had to make on their own, some participants were influenced by opinions from people around them, including relatives and health care professionals. One participant pointed out that her midwife advised against vaccination. Another participant with multiple relatives working in the medical field, trusted their opinion when they explained that vaccination is recommended based on conducted research.

“I discussed it with my family, because they are all doctors. Two GP’s and a neurologist and they said yes, go ahead, it’s safe. We have already seen a lot of studies, I would definitely recommend it” (P4).

Another participant described how the combination of research, media, her partner and his colleagues made her change her mind regarding vaccination.

“Yes, sure, because at first I did not want it […]. Until at one point, the news reported that pregnant women were at high risk, especially towards the end of the last trimester. Your belly is bigger and you have less lung capacity, so the chance is higher that you end up in the ICU. […] I thought: oh, I will soon be heavily pregnant in the winter, during the cold and flu season. […] Colleagues of my husband that are doctors also said: all pregnant women we know, including doctors, had the vaccination themselves. Then I thought: I just have no choice, vaccination is probably safer than I think. The risk of the longer term does not outweigh the actual risk for me and the child that may have to be delivered early if I get COVID” (P2).

One participant was sceptical about the research on which the advice to routinely vaccinate all pregnant women was based on. Another participant explained that she became more doubtful when she came across links on websites that asked her to participate in research regarding COVID-19 vaccination during pregnancy. To her it was a confirmation that vaccination for COVID-19 was still subject of research.

“At first it is discouraged and then strongly recommended after a study in America […]” (P5).

“Especially when I was on the website of the National Institute for Public Health and the Environment (RIVM), links of pregnant women kept popping up: participate in a study so we can see what the vaccine does, and I understand 100%, I fully understand that it is something very normal that this is being investigated. Only with me personally, I do not know, it made me doubt even more, that I thought: oh yes, see, they actually still have to investigate this” (P2).

### 3.3 Societal motivation

The third theme relates to societal motivation and influences to the decision-making process regarding COVID-19 vaccination. Most of the participants spoke about different ways in which COVID-19 influenced society. Participants also reported different ways in which society influenced the decision-making process regarding vaccination. For some participants altruism played a role in the decision to receive vaccination against COVID-19, for other participants external motivation due to additional rules and restrictions for people who were not vaccinated made them decide to get the vaccination.

#### 3.3.1 Altruism

Some participants felt an obligation to consider the consequences for the whole society, in particular vulnerable people and therefore decided to receive vaccination.

“[…] I have not had the feeling that I have to protect myself that much, but […] I just think it is important that as many people as possible get vaccinated in order to prevent this and I wanted to contribute to that myself, so that has been the reason that I just had to get vaccinated” (P8).

#### 3.3.2 External motivation

As previously described, some participants initially did not want to receive vaccination because they were not particularly concerned for their own health and were bothered by the unknown long-term effects. However, due to the Dutch government rules, some participants changed their mind. Requiring a negative COVID-19 test prior to visiting public facilities if not vaccinated was a logistic challenge for participants, especially with a newborn. In addition, the risk of social isolation also provided a new reason to choose vaccination.

“I thought, if the little one is here, and I am up on my feet again, I cannot go anywhere during the winter months, then I would feel very isolated. […]. So now I received my first vaccination two weeks ago and I am going to get the second one next week and then I have a QR code once the baby is here. This is due to the rules that are in place right now […] I felt forced, that sounds heavy, but that is kind of what happened” (P1).

International travel restrictions were also a potential factor influencing the decision. Some of the participants were not originally born in Netherlands and without vaccination unable to visit family in other countries.

“For example Morocco, my parents live there, so if I want to visit my parents, I have to receive the vaccination, because it is mandatory if you want to go there” (P6).

One participant described how she felt judged by society and government for her choice not to vaccinate during pregnancy.

“My vulnerability, so to speak, was completely put aside, as if I did it because I want to be difficult, as if I am a crazy person, it does not matter how you want to call it, but you are not seen nor acknowledged that the position as a pregnant woman is difficult, even aside from all the other factors. I found that very intense” (P7).

## 4 Discussion

### 4.1 Main findings

In this study we explored the barriers and facilitators for pregnant individuals choice and motivation regarding vaccination against COVID-19 during pregnancy in Netherlands.

We found three themes that capture the perspectives of pregnant individuals regarding vaccination against COVID-19: health consequences, ambiguity of information and societal motivation. Health consequences referred to their own health, but also the health and possible consequences for their offspring. Lack of long-term data and therefore uncertainty on possible adverse long-term effects for their offspring, were a main point of concern. In addition, unambiguous information provision based on evidence, with regard to why pregnant individuals were advised to receive vaccination, is an important topic that needs to be addressed. Not only for health care providers, but also for policy makers, national health institutes and societies for maternal and foetal medicine. Furthermore, provided information should also match individuals specific ideas, feelings and perceptions regarding vaccination and in addition take life experiences into account. The unique restrictions resulting from COVID-19 being a pandemic, added societal motivation as a reason for vaccination. Without vaccination there were restrictions to traveling and entering public places. From the societal point of view, altruism was also important and resulted in deciding to get vaccination to protect vulnerable people in society. The three main themes, health consequences, ambiguity of information and societal motivation, indicate that pregnant individuals perspectives for vaccination are shaped by personal experiences and interactions with the broader societal context.

### 4.2 Interpretation of findings

Literature has provided numerous models to explain decision-making in healthcare. In comparison to the 3C model we found that confidence and complacency aligned with the findings from our study. We found a confidence barrier due to safety concern of the vaccine and a complacency barrier regarding not being convinced that contracting a COVID-19 infection negatively impacts their lives. However, our study did not show any convenience barriers, instead we found convenience facilitators such as being able to travel abroad after vaccination and avoiding having to take a COVID test prior to entering public places.

Furthermore, the Health Believe Model (HBM) that is used to explore and explain the rationale behind vaccine hesitancy, also provides similarities to our findings (24, 34). The HBM is based on the hypothesis that the response to a health problem is determined by a persons perceived severity of the threat and the individual perceived susceptibility to the threat. Both of these themes, perceived severity and susceptibility to COVID-19 were also seen in our study. However, in addition to HBM two noteworthy considerations specific for pregnant individuals were identified in our study: (1) the responsibility for their unborn child and (2) the vulnerability to complications from a COVID-19 infection due to the pregnancy.

Although some similarities between our findings and both models are evident, these models do not acknowledge the profound influence of society on individuals decision-making regarding vaccination. A model that would better fit this part of our findings is a conceptual framework of social values in health priority settings, showing the principal of solidarity, described in a few different ways, such as; decisions which give priority to those who are worst-off in health terms which is similar to altruism in our study (35). In addition, the World Health Organization Strategic Advisory Group of Experts on Immunization (SAGE) working group on vaccine hesitancy describes the complex determinants of vaccine hesitancy in three categories: (1) contextual influences, (2) individual and group influences, and (3) vaccine-specific issues (36).

### 4.3 Strengths and limitations

The strength of this study is the study design, a qualitative approach with semi-structured interviews and in-depth questioning, allowing for an open conversation resulting in a broad spectrum of information. We included individuals with variety in maternal ages, parity, educational level, vaccination status, and country of birth, to gain insight from different perspectives, and reached saturation after nine interviews. A team of researchers from various backgrounds and perspectives used interpretative thematic analyses.

One of the limitations of this study is that most participants received care from an academic hospital. This may have introduced selection bias, as academic hospitals predominantly house complicated or high-risk pregnancies which might (unconsciously) influence the decision-making process. Future studies should purposively sample low risk pregnant individuals without a medical or obstetric history. Another possible limitation is that, due to the COVID-19 pandemic, some participants preferred to do the interview by phone or video call, which might have influenced the depth and quality of the interview. In addition, we did not interview any individuals in their first trimester of pregnancy. The first trimester is an important phase in embryonal development and might therefore provide other considerations, like teratogenicity, when it comes to vaccination.

### 4.4 Comparison to other literature

Our results are in line with a previous study from the US showing a paradox regarding foetal wellbeing between vaccinated and unvaccinated pregnant individuals. Whereas unvaccinated individuals were concerned with the paucity of research and potential impact on the development of the foetus and therefore did not receive vaccination, vaccinated pregnant individuals chose vaccination for maternal and foetal protection against possible COVID-19 complications, that might lead to negative effects for the foetus (37). This is similar to our findings, showing that all pregnant individuals thought about consequences for the foetus, but this led to different conclusions on whether or not to choose vaccination.

A previous qualitative study in Turkey has shown similarities, but also differences regarding the opinions of pregnant individuals about COVID-19 vaccines (38). In concordance with our findings pregnant individuals reported that they believe that vaccines may help protect against disease and if recommended by health care professionals this improves the feeling of security. However, in the Turkish study, individuals were afraid for themselves and their babies to die from COVID-19. This is not a particular fear mentioned by pregnant individuals in our study. These differences may be due to general cultural differences and differences in trust in the different health care systems.

Another qualitative study in the United Kingdom (UK), conducted before vaccines became available, highlighted that pregnant individuals perceived a COVID-19 vaccine as riskier than COVID-19 itself (39). This is in line with our findings showing that some pregnant individuals perceived their own health as excellent and were overall worried about the risks and unknown adverse long-term effects of vaccination.

### 4.5 Future recommendations

It is likely that new pandemics will arise in the future, which will also require adaptation and possibly vaccination to prevent illness and spreading of the virus. In addition, there are more infectious diseases, such as respiratory syncytial virus (RSV), for which vaccines are developed to administer during pregnancy (40). However, in recent years trust in vaccination programs has declined, due to doubt about possible side effects and increasing distrust of government organizations in general (41).

Furthermore misinformation through social media is relatively new and should not be underestimated (42).

**Figure 2:**
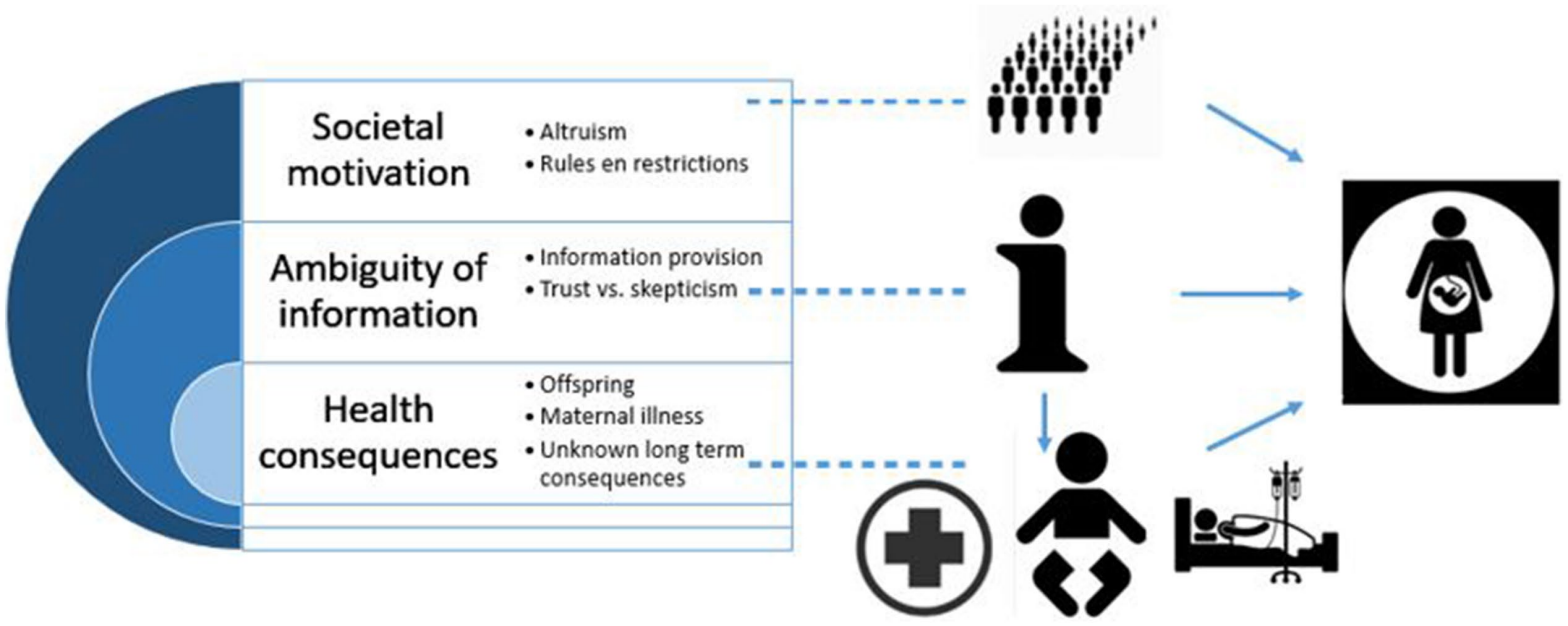
The central focus of the information (i) revolves around three primary themes: societal motivation, ambiguity of information and the health consequences for pregnant individuals all intertwined with each other.

This highlights the importance of providing an open discussion to explore pregnant individuals needs regarding vaccination, enhance knowledge and understanding, and provide specific information to different groups of pregnant individuals, instead of only providing standard information for all pregnant individuals, to reduce vaccine hesitancy (Figure 2).

## 5 Conclusion

Pregnant individuals are a specific and important group when it comes to vaccine hesitancy. They must weigh their own risks and benefits, as well as possible risks and benefits for their unborn child. This study highlighted three main themes that reflect considerations of pregnant individuals in the decision-making process about a new vaccine against COVID-19: health consequences, ambiguity of information and societal motivation. Perceived physical health and information provision regarding the risk of infection during pregnancy and the risks and benefits of vaccination for a pregnant individual and her unborn child are factors that need to be addressed during prenatal consultations discussing vaccination. The different ways in which society influences the decision-making is an important topic for health care workers and policy makers as well.

Our findings can contribute to understand the key points that need to be addressed during conversations between health care providers and pregnant individuals regarding COVID-19 vaccination, and in addition can also be important for future vaccinations during pregnancy. This study underscores the importance of including pregnant individuals in research programs regarding vaccination, considering that the efficiency and adverse effects have a clear impact on successful implementation.

## Data availability statement

The raw data supporting the conclusions of this article will be made available by the authors, without undue reservation.

## Ethics statement

The Medical Ethics Review Committee of VU University Medical Centre examined the study protocol (2021.0245). Official approval was not required. The protocol was in accordance with Dutch privacy regulations. Written informed consent from the patients/ participants to participate in this study was obtained in accordance with the national legislation and the institutional requirements.

## Author contributions

SZ: Data curation, Formal analysis, Investigation, Methodology, Writing – original draft, Writing – review & editing. AR: Conceptualization, Data curation, Methodology, Writing – review & editing, Writing – original draft. NS: Formal analysis, Writing – review & editing, Writing – original draft, Methodology. PB: Conceptualization, Data curation, Formal analysis, Methodology, Supervision, Writing – review & editing, Writing – original draft. BB: Conceptualization, Data curation, Formal analysis, Methodology, Supervision, Writing – review & editing, Writing – original draft. EL: Conceptualization, Data curation, Methodology, Supervision, Writing – review & editing, Writing – original draft. CG: Conceptualization, Data curation, Formal analysis, Investigation, Methodology, Supervision, Writing – review & editing, Writing – original draft.
