# Supplementary material for: Pregnant individuals perspectives towards receiving COVID-19 vaccination during their pregnancy: an in-depth qualitative study
Source: Front Public Health. 2024 Aug 21;12:1415548. doi: 10.3389/fpubh.2024.1415548 (PMC11371620; doi:10.3389/fpubh.2024.1415548)
Supplement: Supplementary file 1 [file Table_1.DOCX]

**Appendix 1.** Interview guide

| Interview guide |
| --- |
| Background questions such as: age, parity, country of birth, educational level, psychological history |
| ‘’What are your general thoughts/ideas about vaccination?’’ |
| ‘’Have you been vaccinated in the past?’’ |
| ‘’Did you receive a Tdap vaccine or a flu vaccine during pregnancy?’’ |
| ‘’What is important to you in the decision making process for vaccination against COVID-19?’’ |
| ‘’Do you have specific fears regarding vaccination?’’ |
| ‘’Do you have personal experience with COVID-19?’’ |
| ‘’What or who are your main sources for information about COVID-19?’’ |
| ‘’What do your relatives/friends/co-workers think about vaccination (during pregnancy)?’’ |
